# Coronary risk factors for the increased of coronary wall thickness : multi-ethnic study of atherosclerosis (MESA)

**DOI:** 10.1186/1532-429X-11-S1-P87

**Published:** 2009-01-28

**Authors:** Cuilian Miao, Shaoguan Chen, Robson Macedo, Shenghan Lai, Kiang Liu, Debiao Li, Steven M Shea, Moyses Szklo, Jens Vogel-Clausen, Joseph Polak, Joao AC Lima, David A Bluemke

**Affiliations:** 1 Johns Hopkins Medicine Institutions, Baltimore, MD USA; 2 Northwestern University Medical School, Chicago, IL USA; 3 Siemens Corporate Research, Inc, Baltimore, MD USA; 4 Tufts University School of Medicine, Boston, MA USA; 5 National Institues of Health, Bethesda, MD USA

**Keywords:** Framingham Risk Score, Coronary Risk Factor, Coronary Calcium Score, Coronary Artery Disease Risk Factor, Arterial Wall Thickness

## Background

Coronary risk factors are established predictors of coronary atherosclerotic disease. The purpose of this study was to investigate the associations of conventional risk factors with increased of coronary artery wall thickness by MR imaging as a marker of atherosclerosis in an asymptomatic population.

## Methods

Coronary wall MR imaging were performed in 194 participants in the Multi-Ethnic Study of Atherosclerosis free of clinical cardiovascular disease (98 men, age 61 ± 9 yrs) using black-blood TSE technique. Cross-sectional coronary artery wall images were acquired and mean wall thickness was determined. Coronary calcium score (CAC) and carotid artery intimal-medial thickness (IMT) were obtained by standard protocols. Linear regression was used to determine the correlations of mean wall thickness and coronary risk factors, the number of risk factors, Framingham risk score, CAC and carotid IMT.

## Results

Male gender, high density lipoprotein (HDL), CAC and number of risk factors were associated with coronary artery wall thickness (p < 0.05). In multivariable analyses, the number of risk factors remained predictive of increased arterial wall thickness (0.0482 mm per risk factor (0.0105, 0.0859), P < 0.05). Framingham risk score and the number of coronary artery disease risk factors were positively related to coronary artery wall thickness in both univariable and multivariable models (p < 0.05) for men only. Wall thickness was significantly greater in men than women (p < 0.05). Large patient size (BMI ≥ 30) limited the ability to detect coronary wall thickness changes by MRI.

## Conclusion

Number of coronary risk factors and Framingham risk score were positively related to coronary artery wall thickness by MRI in asymptomatic men. Significant relationships were not observed in the women, possibly due to small vessel size and/or lower levels of atherosclerosis.

